# Health Worker Compliance with a ‘Test And Treat’ Malaria Case Management Protocol in Papua New Guinea

**DOI:** 10.1371/journal.pone.0158780

**Published:** 2016-07-08

**Authors:** Justin Pulford, Iso Smith, Ivo Mueller, Peter M. Siba, Manuel W. Hetzel

**Affiliations:** 1 Papua New Guinea Institute of Medical Research, Goroka, Papua New Guinea; 2 Liverpool School of Tropical Medicine, Liverpool, United Kingdom; 3 Walter and Eliza Hall Institute of Medical Research, Melbourne, Australia; 4 Barcelona Centre for International Health Research, Barcelona, Spain; 5 Swiss Tropical and Public Health Institute, Basel, Switzerland; 6 University of Basel, Basel, Switzerland; Institut Pasteur, FRANCE

## Abstract

The Papua New Guinea (PNG) Department of Health introduced a ‘test and treat’ malaria case management protocol in 2011. This study assesses health worker compliance with the test and treat protocol on a wide range of measures, examines self-reported barriers to health worker compliance as well as health worker attitudes towards the test and treat protocol. Data were collected by cross-sectional survey conducted in randomly selected primary health care facilities in 2012 and repeated in 2014. The combined survey data included passive observation of current or recently febrile patients (N = 771) and interviewer administered questionnaires completed with health workers (N = 265). Across the two surveys, 77.6% of patients were tested for malaria infection by rapid diagnostic test (RDT) or microscopy, 65.6% of confirmed malaria cases were prescribed the correct antimalarials and 15.3% of febrile patients who tested negative for malaria infection were incorrectly prescribed an antimalarial. Overall compliance with a strictly defined test and treat protocol was 62.8%. A reluctance to test current/recently febrile patients for malaria infection by RDT or microscopy in the absence of acute malaria symptoms, reserving recommended antimalarials for confirmed malaria cases only and choosing to clinically diagnose a malaria infection, despite a negative RDT result were the most frequently reported barriers to protocol compliance. Attitudinal support for the test and treat protocol, as assessed by a nine-item measure, improved across time. In conclusion, health worker compliance with the full test and treat malaria protocol requires improvement in PNG and additional health worker support will likely be required to achieve this. The broader evidence base would suggest any such support should be delivered over a longer period of time, be multi-dimensional and multi-modal.

## Introduction

A global shift in malaria case management (MCM) practice has taken place over the past decade. Healthcare workers in medium- to high- malaria transmission regions are moving away from a presumptive treatment approach in which all cases of febrile illness are treated with antimalarials towards a ‘test and treat’ approach in which new generation antimalarials (artemisinin combination therapies or ACTs) are prescribed following parasitological confirmation by microscopy or rapid diagnostic test (RDT). The test and treat approach reflects current World Health Organisation (WHO) recommendations [1] and has been formally adopted by 96 of 97 countries with ongoing malaria transmission since its introduction [2]. Nevertheless, health worker compliance has often been problematic. In many countries that have adopted the test and treat protocol, fewer than 50% of febrile cases are tested for malaria infection by microscopy or RDT [3–5] and health workers continue to report a lack of confidence in RDT results when they are used [6–10]. Similarly, ACTs are not uniformly prescribed to confirmed malaria cases [11–13] and ACTs or obsolete antimalarials continue to be prescribed presumptively or to malaria RDT negative cases [14–17].

Health worker compliance, according to the ‘systems effectiveness’ model, is one of a number of sequential components that collectively determine the proportion of clinical events that are effectively treated by the formal health care sector [18, 19]. Other components in this model may include (but are not limited to) treatment access, availability of medicines, patient adherence to prescribed medication regimens and the quality of prescribed medications. Studies that have applied this approach to assess the proportion of malaria cases that receive effective treatment within a formal health care sector have reported mixed findings. An overall systems effectiveness of 25% (meaning the formal health care sector effectively cures 25% of all malaria infections) was calculated for children under five years in Zambia, despite relatively high health worker compliance with malaria diagnostic testing (71%) and antimalarial prescription (86%) protocols [20]. A multi-county analysis of Sub-Saharan African countries determined systems effectiveness ranged from as low as 1% in Chad and South Sudan to at most 60% in Botswana, Sao Tome and Principe and Uganda [18]. The relative contribution of health worker compliance to overall systems effectiveness varied greatly between countries, with compliance to firstline antimalarial treatment ranging from less than 10% to greater than 90%, as did the relative contribution of other components within the analysis (e.g. treatment access and patient adherence).

Applications of the systems effectiveness approach in an MCM context (such as those described above) have been criticised for excluding malaria cases that receive antimalarials through non-diagnostic led pathways (e.g. presumptive prescription) and ignoring non-malaria febrile illness (NMFI) cases that receive antimalarials [21]. Accordingly, Rao, Schellenberg and Ghani [21] extended the systems effectiveness model into a decision-tree tool designed to measure a broader array of outcomes, including: correct treatment of malaria with recommended firstline treatment, irrespective of diagnostic pathway (i.e. inclusive of presumptive, clinical or parasitologically confirmed malaria diagnoses); under-treatment of malaria cases; overtreatment of NMFI with firstline antimalarials; and the overall number of febrile patients treated appropriately (both malaria cases given firstline antimalarials and NMFI not treated with firstline antimalarials). Findings from this extended modelling exercise, drawing on data from a number of medium-to-high malaria transmission settings, highlight the complexity, and apparent trade-offs, that occur when modelling systems effectiveness at the broader level of febrile case management as opposed to MCM specifically. For example, provision of 100% stock of ACTs was found to predict a 28.9% point increase in the proportion of malaria cases given an ACT; however, this also resulted in a 26% point increase in the proportion of NMFI cases prescribed an ACT. Thus, when considering MCM alone, 100% ACT stock was beneficial and improved systems effectiveness, yet overall systems effectiveness for the management of febrile illness (inclusive of malarial and non-malarial illnesses) reduced.

The systems effectiveness of MCM or febrile case management has rarely been modelled outside of Africa and never in Papua New Guinea (PNG), a malarious country of approximately 7.3 million people in the Western Pacific. A national survey of health worker compliance conducted in the 12-month period following the introduction of a ‘test and treat’ protocol in PNG produced results illustrative of the potential tension between MCM only and extended febrile case maangement systems effectiveness models [22]. Health worker compliance with the test and treat MCM protocol was relatively high with 68.3% of febrile patients tested for malaria infection by microscopy or RDT and 98.2% of parasitologically confirmed cases prescribed an ACT. Nevertheless, when taking all diagnostic pathways into consideration, only 31.6% of febrile patients prescribed an antimalarial had parasitologically confirmed malaria and only 19.3% of antimalarial prescriptions were ACTs [22]. Thus, a large proportion of febrile cases were still inappropriately treated with antimalarials and the majority of antimalarial prescriptions were for obsolete, ineffective medications.

To better understand health worker compliance with febrile case management treatment practices more broadly, this paper aims to: a) assess health worker compliance on a range of measures relevant to febrile case management and systems effectiveness modelling; b) elucidate self-reported barriers to health worker compliance with current febrile case management protocols; and c) examine health worker attitudes towards current febrile case management protocols. It is anticipated that the resulting findings will provide up-to-date compliance figures and inform locally appropriate interventions to improve health worker compliance.

## Method

This paper presents data from repeat cross sectional surveys of randomly selected primary health care facilities and their respective staff, conducted in the years’ 2012 and 2014. Both surveys were conducted as part of a five year evaluation of the PNG National Malaria Control Program and followed the introduction of the ‘test and treat’ MCM protocol in late 2011. A full description of the evaluation program, including a detailed description of the health facility survey methodology, is presented elsewhere [23].

### Study Setting

PNG is comprised of 22 provinces divided into four regions (Highlands, Momase, Southern and New Guinea Islands) according to their geographical location. A sustained and well-funded National Malaria Control Program has seen a reduction in the general population prevalence of malaria parasitaemia (as measured by light microscopy) to single digit levels [24]. However, 94% of the population are still considered at risk of malaria infection according to the WHO [25]. The PNG Department of Health introduced a test and treat malaria protocol in 2009 [26], although it was not formally implemented until late 2011. Consistent with WHO recommendations [1], the new protocol stipulates that all fever or suspected malaria cases be tested for malaria infection by microscopy or RDT, introduces artemether-lumefantrine (AL) as the new first-line treatment for uncomplicated *Plasmodium falciparum* malaria and AL plus primaquine (PQ) as the new first-line treatment for uncomplicated *Plasmodium vivax* malaria and for mixed malaria infections.

A national health worker training program was carried out in PNG pre-implementation of the test and treat protocol. This program provided detailed instruction on mRDT use and strongly emphasised the importance of restricting antimalarial prescription to test-confirmed cases [27]. A reference manual describing the new protocol in detail was provided to each trainee. ‘Booster’ sessions were provided in some provinces post implementation of the new protocol, although often on an ad-hoc basis and national coverage was not achieved. The training program, reference manual and booster sessions provided relatively little detail on non-malarial febrile case management beyond reference to an Integrated Management of Childhood Illness (IMCI) flowchart. No instruction on the management of non-malarial febrile illness in adolescent or adult patients was provided. The PNG National Department of Health and the Paediatric Society of PNG issues standard treatment guidelines which outline recommended management of a range of common illnesses, including malaria and non-malarial febrile illness [28, 29]. No other training, resources or support have been specifically provided to assist the PNG healthcare workforce comply with the test and treat protocol or manage the increasing number of non-malarial febrile illness cases.

The majority of health services in PNG are delivered through government- and church-providers via an extensive health facility network. This network comprises seven levels of service provision including (in ascending order from lowest level of care to highest): Level 1 (Aid Post), Level 2 (Health Sub-Centre), Level 3 (Health Centre), Level 4 (District Hospital), Level 5 (Provincial Hospital), Level 6 (Regional Hospital) and Level 7 (National Referral Hospital). Level 1–3 facilities largely offer primary health care services only, although inpatient admission is possible in some Level 2 and 3 facilities for low risk procedures and/or illnesses. The PNG health workforce largely consists of (in descending order from highest to lowest qualification): Medical Doctors, Health Extension Officers, Nurses, Community Health Workers (CHWs) and Rural/Medical Laboratory Assistants. A Level 1 facility is typically staffed by a single CHW, whereas an average of 10 health workers (typically CHWs and nurses) are employed at Level 2–3 facilities [30].

### Study Sample

The study sample for each cross sectional survey consisted of up to six primary health care facilities (Levels 1–3) from each of 20 PNG provinces in 2012 and each of 10 provinces in 2014, using a simple random sampling procedure repeated for each survey (sampled provinces listed in S1 Table). The sampling frame was a list of all operational primary health care facilities in participating provinces as provided by the PNG Department of Health.

### Procedure

The 2012 survey was carried out from June to November and the 2014 survey from September to December. A two-to-three member research team spent between one-to-five days at each participating health facility (more time spent at larger facilities, less time at smaller facilities). The research team completed a range of survey instruments at each site, including an audit of supplies, non-participant observation of febrile case management and health worker and patient interviews. Data presented in this paper is limited to that obtained from the non-participant observations of febrile case management and structured, interviewer-administered questionnaires completed with consenting health workers. Oral informed consent was sought from the officer in charge at all participating health facilities and from all participating patients and health workers prior to observation/interview. Patients were considered eligible for participation if they were outpatients, presenting with febrile symptoms or reported a recent history of fever, had not been treated for malaria infection in the past 14 days and were sent home at the end of their consultation (to exclude severe cases of malaria infection from the analyses). Eligible patients were identified upon first contact with a health worker or, if circumstances allowed, by screening in the waiting area prior to first contact with a health worker. Health workers were considered eligible for participation if they held a formal health worker qualification, if they reported treating febrile patients as part of their routine clinical duties and if the test and treat protocol had been implemented in their respective health facility at the time of interview.

### Ethics Statement

The study was approved and granted ethical clearance by the Medical Research Advisory Committee (MRAC) of PNG (MRAC No. 10.12; 26 Feb 2010). The PNG MRAC approved the use of oral consent rather than written consent as the study took place in the frame of routine monitoring and evaluation of the PNG National Malaria Control Program conducted on behalf of the National Department of Health. Written consent was not considered appropriate in this context. The provision of oral consent was recorded on all participant questionnaires/observation checklists. Oral consent was obtained from caregivers/guardians on behalf of minors/children enrolled in the study.

### Measures

The non-participant observation of febrile case management was undertaken by a trained research officer who passively observed the management of fever patients from the point of initial contact with a health professional until service exit or admission onto a treatment ward. During the course of this observation, the research officer recorded whether specified actions did or did not occur as well as the content of specific actions (e.g. whether an antimalarial was prescribed and, if yes, what type of antimalarial) on a structured checklist. The checklist was divided into discrete sections including diagnosis, prescription and treatment counselling and was informed by input from experienced medical- and medical research- professionals. The health worker interview consisted of open and closed questions pertaining to education, work experience and supervision, type and utility of any malaria-related training received (inclusive of training on new treatment policy), knowledge, attitudes and practices relevant to malaria case management and experiences implementing the new treatment policy.

### Data Analysis

All data were double entered into DMSys version 5.1 (Sigma Soft International). Stata/SE version 12 was used for statistical analysis. Univariate analysis was performed to describe the characteristics of the various samples and for calculating 95% confidence intervals (CIs) on selected measures. Differences on repeat measures across time were examined by chi-square or two-tailed *t*-tests as appropriate. The calculation of all CIs was adjusted for possible clustering at the health facility level by using the Stata ‘svy’ command in which health facilities were defined as the primary sampling unit.

Patients were only included in the analyses for the non-participant observation sample if they attended a health facility that had RDTs or operational microscopy and AL and PQ in stock. Thus, the necessary resources to comply with the test and treat protocol were available in all cases. An antimalarial prescription was considered correct (i.e. compliant with protocol) if: AL was prescribed to *P*. *falciparum* cases; AL + PQ was prescribed to *P*. *vivax* or mixed malaria infection cases; or if AL or AL + PQ was prescribed to any malaria ‘positive’ case in which the species type was not identified. The response options for each ‘attitudinal’ statement were ‘agree’, ‘disagree’ and ‘don’t know’. ‘Don’t know’ responses were categorised as ‘incorrect’ in the analysis.

## Results

### Study Samples

#### Non-participant observations

The case management of 771 febrile patients from 56 primary healthcare facilities met the inclusion criteria. Three healthcare facilities were sampled in both surveys. All other healthcare facilities were included in one survey only (37 in 2012 and 22 in 2014). Table 1 presents selected patient characteristics by survey year as well as the location of the respective health facility in which treatment was observed. As shown, a majority of participants were from the Momase (34.5%) or Southern (24.4%) regions. Participation was relatively evenly split by sex and age grouping.

**Table 1:** Location and selected characteristics of the febrile patient sample.

Characteristic		2012 (n = 395)	2014 (n = 376)	Overall (n = 771)
Location	Southern	32.4%	16.0%	24.4%
	Highlands	20.8%	16.0%	18.4%
	Momase	30.4%	38.8%	34.5%
	Islands	16.4%	29.2%	22.7%
Sex	Male	46.6%	53.2%	49.8%
	Female	53.4%	46.8%	50.2%
Age	<5 yrs	41.4%	36.8%	39.2%
	5–14 yrs	23.0%	25.3%	24.1%
	15+ yrs	35.6%	37.9%	36.7%

#### Health worker interviews

A total of 265 health workers from 88 primary healthcare facilities met the inclusion criteria. Four healthcare facilities were sampled in both surveys. All other healthcare facilities were included in one survey only (a total of 46 in each year). Table 2 presents selected health worker characteristics by survey year as well as the location of their respective health facilities. A majority of participants were from the Momase (34.3%) or Southern (28.7%) region, were female (56.2%) and held a CHW qualification (64.9%). The mean age of participants was 40.1 years (SD 10.1) and the mean number of years in employment was 17.5 (SD 11.2).

**Table 2:** Location and selected characteristics of the health worker sample.

Characteristic		2012 (n = 153)	2014 (n = 112)	Overall (n = 265)
Location	Southern	39.9%	13.4%	28.7%
	Highlands	19.6%	18.8%	19.3%
	Momase	21.6%	51.8%	34.3%
	Islands	18.9%	16.0%	17.7%
Sex	Male	41.8%	46.4%	43.8%
	Female	58.2%	53.6%	56.2%
Qualification	Dr	0.7%	0%	0.4%
	HEO	3.3%	6.3%	4.5%
	Nurse	26.1%	29.8%	29.8%
	CHW	69.2%	64.9%	64.9%
	Pharmacist	0.7%	0%	0.4%
Age (in years)		39.9 (SD 10.0)	41.8 (10.2)	40.1 (SD 10.1)
Work experience (in years)		16.2 (SD 11.5)	19.3 (SD 10.7)	17.5 (SD 11.2)

### Compliance

#### Observed compliance

Table 3 presents the total number and type of antimalarial prescriptions (none, correct or incorrect) by malaria diagnostic test result (no test, malaria positive, malaria negative) per survey year. Out of a total of 169 incorrect antimalarial prescriptions, 78.1% (132/169) were due to the exclusive use of non-recommended antimalarials (e.g. chloroquine), 17.2% (29/169) were due to a failure to combine PQ with an AL prescription when treating a non-*P*. *falciparum* malaria infection, 2.4% (4/169) were for combining PQ with AL when treating a *P*. *falciparum* infection and a further 2.4% (4/169) were for combining AL with an obsolete antimalarial. A full description of the types of antimalarial prescription by diagnostic test result is presented in S2 Table.

**Table 3:** Frequency of antimalarial prescription by diagnostic test result.

Diagnostic Test Result	Antimalarial Prescription							
	2012				2014			
	N	Nil	Correct	Incorrect	N	Nil	Correct	Incorrect
No test	102	48	4	50	71	65	0	6
Malaria +	53	0	36	17	69	0	44	25
Malaria -	240	193	1	46	236	210	1	25
Total	395	241	41	113	376	275	45	56

The range of health worker compliance indicators, as informed by data in Table 3, are listed in Table 4. As shown, 74.2% (53+240/395) of current/recent febrile patients received a malaria diagnostic test in 2012 as compared to 81.1% (69+236/376) in 2014, a statistically significant increase (*x*^2^(1) = 5.33, p = 0.021). Of those patients with confirmed malaria, 67.9% (36/53) received the correct antimalarial/s in 2012 as compared to 63.8% (44/69) in 2014 (*x*^2^(1) = 0.23, p = 0.632). All confirmed malaria cases were prescribed an antimalarial (i.e. no under treatment was evident). When all diagnostic pathways are taken into account (i.e. a presumptive, clinical or confirmed diagnosis of malaria), the correct antimalarial was prescribed in 26.5% (41/41+113) and 45.0% (45/45+56) of malaria cases in 2012 and 2014, respectively. This increase reached statistical significance (*x*^2^(1) = 9.14, p = 0.002). The percentage of current/recent febrile patients prescribed an antimalarial despite testing negative for malaria infection decreased, to a statistically significant level, from 20.0% (1+46/240) in 2012 to 10.6% (1+25/236) in 2014 (*x*^2^(1) = 9.02, p = 0.011). Compliance with prescription protocols irrespective of diagnostic pathway (i.e. prescribing the recommended antimalarial/s to patients diagnosed with presumptive, clinical or confirmed malaria and not prescribing any antimalarial/s to NMFI patients) increased from 70.9% (48+4+36+193+1/395) to 85.1% (65+44+210+1/376) over the same time period. Compliance with both diagnostic and prescription protocols increased from 58.0% (36+193/395) to 67.8% (44+210/376). Increases in these last two measures of compliance reached statistical significance (*x*^2^(1) = 22.57, p<0.001 & *x*^2^(1) = 7.99, p = 0.005, respectively).

**Table 4:** Compliance indicators.

Compliance Indicator	2012	2014	Overall
	% (95% CI)	% (95% CI)	% (95% CI)
Febrile patients tested for malaria infection by RDT or microscopy	74.2% (58.7, 85.3)	81.1% (65.0, 90.8)	77.6% (67.2, 85.4)
Confirmed malaria cases prescribed recommended antimalarial/s	67.9% (46.9, 83.5)	63.8% (49.6, 79.5)	65.6% (54.2, 75.4)
Confirmed malaria cases not prescribed any antimalarial	0%	0%	0%
Presumptive/clinical/confirmed malaria cases prescribed recommended antimalarial/s^a^	26.5% (12.9, 46.6)	45.0% (35.8, 54.5)	33.7% (22.9, 46.6)
Confirmed non-malaria febrile illness cases prescribed an antimalarial	20.0% (9.7, 36.7)	10.6% (5.6, 19.2)	15.3% (9.2, 24.5)
Compliance with prescription protocols irrespective of diagnosis type^b^	70.9% (55.1, 82.9)	85.1% (78.0, 90.2)	77.8% (68.7, 84.9)
Compliance with diagnostic and prescription protocols^c^	58.0% (43.6, 71.2)	67.8% (55.7, 77.9)	62.8% (53.4, 71.3)

a. A presumptive malaria case is defined as any patient prescribed the recommended firstline antimalarial/s, despite no confirmatory diagnosis. A clinical malaria case is defined as any patient prescribed the recommended firstline antimalarial/s, despite testing negative for malaria via a diagnostic test.

b. Presumptive/clinical/confirmed malaria cases given the recommended firstline antimalarial/s and NMFI not treated with antimalarials. In this calculation, presumptive and clinical malaria diagnoses in which the recommended firstline antimalarial/s is prescribed are assumed to be correct.

c. In this calculation, presumptive and clinical diagnosis of malaria are assumed to be incorrect (non-compliant with protocol)

In addition, 21.3% (95% CI 11.8, 35.6) of febrile patients were advised to sleep under a mosquito net in 2012 as compared to 20.4% (95% CI 8.0, 43.1) in 2014 and 21.0% (95% CI 13.0, 32.1) overall. This difference between time-periods failed to reach a level of statistical significance (*x*^2^(1) = 0.23, p<0.632).

#### Self-reported compliance

80.8% (214/265) of health workers reported managing at least one febrile patient in the two weeks prior to survey. The total number of febrile patients reportedly managed was 5856 (2012, n = 3319; 2014, n = 2537) at a median of 15 (IQR 22) patients per participant (the comparative figures for 2012 and 2014 were 18.5 (IQR 32) and 12 (IQR 18), respectively). According to health worker self-report, 65.9% of these patients were tested for malaria infection by RDT or microscopy (58.2% & 76.0% in 2012 and 2014, respectively), 34.6% were prescribed an antimalarial (28.4% & 42.6%, respectively) and 27.7% were provided malaria prevention advice (23.0% & 33.9%, respectively).

Table 5 presents the percentage of health workers who reported at least one instance of non-compliance in the two weeks prior to survey. Overall, health workers were most likely to report at least one instance of failing to provide malaria prevention advice (89.7% of the total), followed by failing to test for malaria infection by RDT or microscopy (40.2%), providing an incorrect antimalarial prescription (32.3%) and providing an antimalarial to a RDT negative patient (23.7%). The variations observed in the percentage of health workers reporting at least one instance of non-compliance across the two time periods reached a level of statistical significance on only one measure: failing to test for malaria infection by RDT or microscopy which decreased from 46.7% in 2012 to 31.9% in 2014 (*x*^2^(1) = 4.77, p = 0.029).

**Table 5:** Percentage of health workers self-reporting at least one instance of non-compliance in the two weeks prior to survey.

Form of Non-Compliance	% Self-Reporting (95% CI)^a^		
	2012	2014	Overall
	(n = 120)	(n = 94)	(n = 214)
Failed to test for malaria infection by RDT/MS	46.7 (35.1, 58.6)	31.9 (20.6, 45.8)	40.2 (31.6, 49.5)
Provided incorrect antimalarial prescription^b^	37.1 (23.4, 53.2)	26.4 (14.7, 42.8)	32.3 (23.0, 43.2)
Provided an antimalarial prescription to a RDT negative patient ^C^	23.9 (14.0, 37.7)	23.5 (13.7, 37.2)	23.7 (16.0, 33.6)
Failed to provide malaria prevention advice	88.3 (76.8, 94.5)	91.5 (84.1, 95.6)	89.7 (83.7, 93.7)

a, Self-reporting non-compliance with at least one febrile/recently febrile patient in the two weeks prior to survey.

b, Analysis limited to those participants who reported providing at least one antimalarial prescription in two weeks prior to survey (n = 161).

C, Analysis limited to those participants who reported at least one malaria negative patient as confirmed by RDT or microscopy in the two weeks prior to survey (n = 190).

### Self-Reported Barriers to Compliance

Tables 6 and 7 present self-reported reasons why participating health workers did not test at least one febrile/recently febrile patient for malaria infection by RDT in the two weeks prior to survey or why AL was not prescribed (when antimalarial prescriptions were given). The most widely reported reason for not conducting a RDT was the absence of fever at the time of diagnosis or perceived absence of other common malaria symptoms. Nil or low RDT stock was the next most widely reported reason. Five participants reported that it was not their duty to complete RDTs and that they expected the patient to receive the diagnostic test at another point during the clinical consultation.

**Table 6:** Frequency of self-reported reasons for not using a RDT.

Reasons	2012	2014	Overall
	(n = 37)	(n = 27)	(n = 64)
Absence of fever/malaria symptoms	22	19	41
Nil/low RDT stock	6	4	10
RDT completed elsewhere	1	4	5
Inadequate training	3	0	3
Patient refused test	2	0	2
Don’t use RDT at first presentation	2	0	2
Too busy	1	0	1

**Table 7:** Frequency of self-reported reasons for prescribing non-recommended antimalarial medication.

Reasons	2012	2014	Overall
	(n = 21)	(n = 10)	(n = 31)
Recommended medication reserved for RDT+ cases	16	5	21
Clinical judgement	3	1	4
Recommended medication out of stock	1	3	4
Preserve limited supply of recommended medication	0	1	1
Deplete existing supply of obsolete medication	1	0	1

The most frequently reported reason for not prescribing the correct antimalarial medication was that the recommended firstline antimalarial medications were thought to be reserved for RDT postive cases only. In other words, when health workers chose to prescribe an antimalarial to a RDT negative patient or a patient who had not received a RDT they often believed it was inappropriate to prescribe the recommended firstline antimalarial regimen in these circumstances and opted for obsolete antimalarials instead.

The most frequently reported reason for prescribing an antimalarial medication to at least one RDT negative patient in the two weeks prior to survey was a clinical diagnosis of malaria despite the negative RDT result (n = 33), followed by patient insistence (n = 1) and as a prophylactic measure (n = 1). The most frequently reported reason for not providing malaria prevention advice to at least one febrile/recently febrile patient in the two weeks prior to survey was “too busy or insufficient time” (n = 32), followed by “forgot” (n = 7) or a belief that the provision of such advice was unnecessary (n = 7).

Health workers were further asked if they had experienced any problem(s) implementing the test and treat malaria protocol. Overall, 44.4% (95% CI 37.3, 51.7) of health workers reported that they had experienced at least one problem, with a modest and statistically insignificant increase across time periods: 43.2% (95% CI 34.4, 52.6) and 46.0% (95% CI 35.2, 57.1) in 2012 and 2014, respectively (*x*^2^(1) = 0.19, p = 0.665). The most frequently reported problem was perceived knowledge gaps in understanding the full protocol (n = 55), followed by a reported lack of confidence in one or more aspects of the new protocol (n = 27), typically the perceived reliability of RDT results or perceived effectiveness of AL. Shortages of either RDTs or AL were the third most frequently reported problem (n = 24).

### Health Worker Attitudes towards the Test and Treat Protocol

Health workers were presented with nine statements designed to measure attitudinal support for the test and treat protocol. Table 8 lists the nine statements, the ‘correct’ response (i.e. a response considered supportive of the test and treat protocol) for each statement and the percentage of participants who responded correctly. As shown, the percentage of participants responding correctly increased on seven out of the nine statements between 2012 and 2014 and decreased on two (‘advising patients how best to avoid mosquito bites is a good use of clinical time’ and ‘telling patients when to take their medication is less important if written instructions are provided’). The reported changes in the percentage of participants who provided a correct response reached statistical significance on two statements: “All patients who present with fever or suspected malaria should be tested for malaria infection by microscopy or RDT” (90.2% & 98.2% in 2012 & 2014, respectively; *x*^2^(1) = 6.92, p = 0.009); and “In most cases, combination therapy is the most effective treatment for malaria infection” (51.6% & 67.0% in 2012 & 2014, respectively; *x*^2^(1) = 6.24, p = 0.012). Overall, the mean number of correct statements (out of nine) was 6.8 (SD 1.5). The mean number of correct statements in 2012 and 2014 were 6.6 (SD 1.5) and 7.1 (SD 1.4), respectively; a statistically significant increase, *t*(262) = -2.961, p = 0.003.

**Table 8:** Health worker responses to nine attitudinal statements.

Statement	‘Correct’ Response	% (95% CI) of Participants’ Providing Correct Response		
		2012	2014	Overall
All patients who present with fever or suspected malaria should be tested for malaria infection by microscopy or RDT	Agree	90.2 (83.8, 94.3)	98.2 (93.4, 99.5)	93.6 (89.6, 96.1)
In most cases, chloroquine is an effective treatment for uncomplicated malaria infection	Disagree	47.7 (39.1, 56.4)	55.4 (43.8, 66.4)	50.9 (44.3, 57.6)
Advising patients how best to avoid mosquito bites is a good use of clinical time	Agree	77.8 (70.7, 83.6)	76.8 (64.4, 85.8)	77.4 (71.1, 82.6)
In most cases, clinical diagnosis is just as accurate as microscopy or RDT in detecting malaria infection	Disagree	68.6 (57.3, 78.1)	77.7 (68.3, 84.9)	72.5 (64.9, 78.9)
Fever patients who test negative for malaria infection should still be provided with antimalarial medication as a precautionary measure	Disagree	68.0 (56.9, 77.4)	74.1 (63.4, 82.6)	70.6 (62.7, 77.4)
It is important to distinguish between vivax and falciparum infection when treating uncomplicated malaria	Agree	79.7 (72.6, 85.4)	87.4 (78.2, 93.0)	83.0 (77.8, 87.1)
Telling patients when to take their medication is less important if written instructions are provided	Disagree	84.3 (78.2, 89.0)	83.9 (76.3, 89.4)	84.2 (79.7, 87.8)
In most cases, combination therapy is the most effective treatment for malaria infection	Agree	51.6 (42.2, 60.9)	67.0 (57.3, 75.4)	58.1 (51.1, 64.8)
Malaria patients are less likely to complete their medication if the importance of doing so is not clearly communicated to them	Agree	89.5 (83.4, 93.6)	91.1 (83.9, 95.2)	90.2 (85.9, 93.3)

Overall, 87.0% (95% CI 77.4, 92.9) of health workers reported that malaria treatment had improved as a result of the test and treat protocol. An increase was evident across time-periods with 84.0% (95% CI 67.9, 92.9) and 91.1% (95% CI 79.6, 96.4) of health workers reporting improvement in 2012 and 2014 (respectively), although this did not reach statistical significance (*x*^2^(1) = 2.84, p = 0.092). The most frequently reported reason for improvement was a perceived increase in the accuracy of malaria diagnosis with the introduction of RDTs (n = 110). This was followed by a perceived improvement in treatment outcome as a result of the new antimalarial (n = 84) and a perceived reduction in malaria cases (n = 65). The latter was reported as either a result of fewer malaria cases in the community or as a result of more accurate diagnosis (meaning fewer malaria cases were recorded).

## Discussion

Compliance with core MCM ‘test and treat’ protocols was observed in the majority of cases with 77.6% of patients tested for malaria infection by RDT or microscopy and 65.6% of confirmed malaria cases prescribed the correct antimalarial/s (and all confirmed malaria cases provided an antimalarial of some description). In addition, only 15.3% of febrile patients who tested negative for malaria infection were subsequently prescribed an antimalarial. Encouragingly, statistically significant improvements were identified on all but one of the compliance measures between 2012 and 2014, indicating that PNG health workers have become increasingly compliant with the test and treat protocol in the two-to-three year period immediately following its introduction. These figures compare favourably with health worker compliance rates internationally [3–5, 15, 31, 32].

Observed compliance with recommend antimalarials was less impressive when all diagnostic pathways were considered (i.e. inclusion of presumptive/clinical malaria diagnosis), with only 33.7% of patients diagnosed with malaria by any means provided the correct antimalarial. This finding indicates that PNG primary healthcare workers prescribe less effective, obsolete antimalarials to presumptively/clinically diagnosed malaria patients as compared to confirmed malaria cases, a practice recorded elsewhere [15, 32]. The majority of those health workers who reported prescribing an incorrect antimalarial stated as much, with 67.7% choosing to reserve recommended firstline antimalarials for confirmed malaria cases only. Similarly, while overall compliance with prescription protocols was relatively high when diagnostic pathway was not considered (77.8%), compliance with a strictly defined ‘test and treat’ protocol (all febrile cases tested by RDT or microscopy, correct antimalarial only prescribed to confirmed malaria cases, no antimalarial prescription to test negative cases) was much lower (62.8%). These findings suggest that there is still considerable room for improvement in health worker compliance in routine diagnostic testing and antimalarial prescribing practices and support the contention by Rao et al that systems effectiveness modelling based on strict MCM parameters may obscure limitations in broader febrile case management practice [21].

Health worker attitudes towards the test and treat protocol were largely positive with 84.0% of participants reporting MCM to have improved as a result of the new test and treat protocol in 2012, increasing to 91.1% in 2014. Similarly, on a nine-item measure designed to assess attitudinal support for the test and treat protocol, the majority of responses were ‘correct’ (or supportive) at both time-points and the mean number of ‘correct’ responses improved over time. The attitudinal data reflect the relatively high and increasing health worker compliance with febrile case management protocols. What is uncertain at this stage is whether health worker compliance in PNG will increase further with continued exposure to the test and treat protocol, whether health worker compliance is now at or approaching a maximum level achievable given current (limited) health worker supports or whether these initial compliance gains may deteriorate over time. In support of the latter two possibilities was the finding that a consistent and not in-significant percentage of participants reported at least one instance of non-compliance in the two weeks prior to survey in both 2012 and 2014. A large number of barriers to compliance were identified by health workers, but four in particular were widely reported. These included: a reluctance to test current/recently febrile patients for malaria infection by RDT or microscopy in the absence of acute malaria/fever symptoms; reserving recommended firstline antimalarials for confirmed malaria cases only; choosing to clinically diagnose a malaria infection, despite a negative RDT result; and having too little time to provide malaria prevention advice. In addition, just under half of the health worker sample (44.4%) reported experiencing at least one problem implementing the test and treat protocol, a percentage that increased slightly across time (43.2% vs. 46.0%, respectively). The most frequently reported problem was a perceived knowledge gap in understanding the full protocol and a lack of confidence in one or more aspects of the protocol, which was typically the perceived reliability of RDT results.

The reported reluctance to use a RDT with recently febrile patients who do not present with overt symptoms of malaria infection as well as a continued distrust in RDT negative results are issues of particular concern. For while health worker compliance with RDT use increased over the two survey periods, and antimalarial prescription decreased, it is possible that these trends may reverse in a context of declining malaria transmission as is the case in PNG [24]. As fewer and fewer current/recently febrile patients present to health services, or test positive for malaria infection when they do, then the utility of routine RDT testing may become less apparent and compliance with diagnostic protocols may decline. Health worker distrust of RDT negative results has been widely documented internationally [6–10] and is likely exacerbated by a dearth of cheap and reliable diagnostic point of care tests for other febrile illnesses [33]. In the absence of a reliable means of differential diagnosis, or intensive training to support differential diagnosis, then health workers may revert to a previous practice of clinically diagnosing malaria or they may adopt other ‘convenience’ diagnoses and prescription practices, as may already be evident by high rates of antibiotic prescription in RDT negative cases [34–36]. Adhering to RDT results has been proven safe in the PNG context [37], so the continued distrust suggests further clinical education is warranted. The current practice of reserving recommended firstline antimalarials for confirmed malaria cases is perhaps less concerning as it is more amenable to correction through drug procurement and supply mechanisms. If the supply of obsolete antimalarials is systematically decreased then the prescription of recommended antimalarials can only increase (at least relative to other prescriptions and assuming a consistent or increasing supply) as has previously been observed [3]. Having said this, availability of recommend antimalarials is not in itself sufficient to ensure correct prescription practice [3, 38].

A main aim of this study was to identify potential interventions to improve health worker compliance with the test and treat malaria protocol in Papua New Guinean primary health care facilities. Findings from this study suggest the greatest gains in health worker compliance would be achieved by (in order of importance): promoting uniform prescription compliance with recommended firstline antimalarials irrespective of whether a patient is diagnosed with malaria presumptively, by RDT or microscopy or clinically despite a negative RDT result; promoting routine testing for malaria infection by RDT or microscopy for all febrile or recently febrile patients irrespective of the decreasing malaria burden in PNG; and discouraging the prescription of antimalarials to RDT negative patients who are not clinically suspected of severe malaria, at least during an initial clinical consultation. How best to increase health worker compliance in these areas remains an open question. A review of health worker support interventions in low resource settings concluded that multiple means of support are almost always necessary to improve health worker performance [39]. Available evidence largely supports this contention in a malaria-specific context. Short-term or one-off interventions designed to improve MCM often produce limited or mixed results [40–42], while an increasing number of studies have demonstrated statistically significant and sustained improvement in MCM through longer-term health worker support interventions. These include: a two day training, multiple interactive workshops, and supportive SMS collectively delivered over a 14-month period [43]; a two-three day training, regular on-site clinical mentorship and supportive SMS collectively delivered over a 6-month period [44]; a three week ‘integrated management of infectious disease’ training, followed by two one week booster sessions (12- and 24-weeks post training) and on-site support two days a month for a nine-month period [45]; and supportive SMS sent over a six-month period [46]. Thus, a longer-term health worker support program, possibly consisting of varied components, may be most likely to produce significant and sustained change in health worker malaria/febrile case management practice in PNG. Consistent with a previous recommendation by Galactionova et al [18], greater gains in overall health worker compliance may also be achieved if the focus of any such intervention is on all components of MCM (e.g. diagnostic, prescription and treatment counselling practices) rather than a specific aspect (e.g. non-prescription of antimalarial medication to RDT negative patients).

The findings reported in this study are also notable for the remarkable similarity between observed and self-reported compliance figures. Health workers self-reported testing 65.9% of febrile patients for malaria infection by RDT or microscopy, prescribing an antimalarial to 34.6% of febrile patients and giving malaria prevention advice to 27.7%. The comparative figures for observed (unmatched) febrile patients were 77.6%, 33.7% and 21.0%, respectively. Health worker self-report could be, therefore, a potentially reliable proxy indicator of MCM practice and possibly more accurate than patient self-report. Patient self-report has previously been found to be a reasonable proxy measure of diagnostic and prescription MCM practice in PNG, but a poor proxy measure of treatment counselling (e.g. the provision of malaria prevention advice) [47]. The sensitivity and specificity of health worker self-report as a proxy measure of MCM would need to be established before reliance conclusions can be drawn; the data presented in this paper suggest this may be an exercise worth exploring.

The reported study was not without limitation. Participating clinicians were aware that they were being observed and may have altered their clinical practice accordingly. The expected effect of any such bias would be towards perceived ‘better’ practice (i.e. higher compliance). Similarly, health workers may have been reluctant to self-report non-compliance, or at least the full extent of non-compliance, in response to interview questions. Nevertheless, the high degree of convergence between observed and self-reported measures of compliance improves confidence in the reported findings. The reported compliance figures were based on medication type only and could potentially be lower if drug dosage had been taken into consideration. Finally, the sample excluded secondary-care and private-sector health facilities and may not be representative of malaria case management in these settings.

### Conclusion

Health worker compliance with a test and treat malaria protocol is higher in PNG relative to other malarious countries at a similar stage of protocol implementation and has improved significantly in the two-to-three year period following its introduction. Nevertheless, compliance was far from perfect on a number of measures and the level of compliance varied depending on whether strict MCM criteria were applied or not. The study identified a small number of persistent barriers to health worker compliance and the findings raise the possibility that health worker compliance with the test and treat protocol may now be at a maximum threshold or may even decline without further intervention. Key focal areas for intervention should include the uniform prescription of recommended firstline antimalarials irrespective of diagnostic pathway, routine testing for malaria infection by RDT or microscopy for all febrile or recently febrile patients and the non-prescription of antimalarials to RDT negative patients. The broader evidence base would suggest any such intervention be delivered over a longer period of time (e.g. six months or more), be multi-dimensional (e.g. focus on all three areas, rather than any one) and multi-modal.

## Supporting Information

S1 Table Sampled provinces by survey year. (DOCX)

S2 Table Antimalarial prescription type by diagnostic test result. (DOCX)
