# Supplementary material for: Health Worker Compliance with a ‘Test And Treat’ Malaria Case Management Protocol in Papua New Guinea
Source: PLoS One. 2016 Jul 8;11(7):e0158780. doi: 10.1371/journal.pone.0158780 (PMC4938505; doi:10.1371/journal.pone.0158780)
Supplement: S1 Table — (DOCX) [file pone.0158780.s001.docx]

S1 Table. Sampled provinces by survey year

| **2012** | **2014** |
| --- | --- |
| Western | Central |
| Gulf |  |
| Central |  |
| National Capital District |  |
| Milne Bay |  |
| Northern | Northern |
| Southern Highlands Province |  |
| Enga |  |
| Western Highlands Province |  |
| Chimbu | Chimbu |
| Eastern Highlands Province | Eastern Highlands Province |
| Morobe | Morobe |
| Madang | Madang |
| East Sepik | East Sepik |
| West Sepik | West Sepik |
| Manus |  |
| New Ireland Province | New Ireland Province |
| East New Britain | East New Britain |
| West New Britain |  |
| Autonomous Region Of Bougainville |  |
