# Supplementary material for: Health Worker Compliance with a ‘Test And Treat’ Malaria Case Management Protocol in Papua New Guinea
Source: PLoS One. 2016 Jul 8;11(7):e0158780. doi: 10.1371/journal.pone.0158780 (PMC4938505; doi:10.1371/journal.pone.0158780)
Supplement: S2 Table — (DOCX) [file pone.0158780.s002.docx]

S2 Table. Antimalarial prescription type by diagnostic test result

| **Diagnostic Test Result** | | **Prescription** | | | | |
| --- | --- | --- | --- | --- | --- | --- |
|  |  | Rating | N | 2012 | N | 2014 |
| No test |  | Correct | 4 | AL | 0 |  |
|  |  | Incorrect | 50 | CQ+SP(x34), AQ+SP(x9), AQ(x3), SP(x1), Q+SP(x1), PQ+A+SP(x1), AL+A+SP(x1) | 6 | SP(x3), AQ+SP(x1), CQ+SP(x1), AT(x1) |
| Malaria + | Undefined | Correct | 3 | AL | 1 | AL |
|  |  | Incorrect | 0 |  | 1 | Q |
|  | *P.falciparum* | Correct | 18 | AL | 17 | AL |
|  |  | Incorrect | 1 | AL+PQ | 5 | AL+PQ(x3), A+PQ(x1), A(x1) |
|  | *P.vivax, non-P.f* | Correct | 7 | AL+PQ | 3 | AL+PQ |
|  |  | Incorrect | 2 | AL | 7 | AL |
|  | Mixed infection | Correct | 8 | AL+PQ | 23 | AL+PQ |
|  |  | Incorrect | 14 | AL(x13), AL+PQ+AQ | 12 | AL(x6), AL+A(x3), A(x3), Q(x1) |
| Malaria - |  | Correct | 1 | AL | 1 | AL |
|  |  | Incorrect | 46 | CQ+SP(x19), AQ+SP(x14), AQ(x5), A+SP(x3), A(x2), SP(x1), CQ+SP+PQ(x1), AT+SP(x1) | 25 | SP(x7), CQ+SP(x5), AQ+SP(x3), PQ(x3), A(x2), Q(x2), A+SP(x1), CQ+Q(x1), AT(x1) |

A=artemether tablets; AL= artemether-lumefantrine; AT=artesunate tablets; AQ=amodiaquine; CQ=chloroquine; Q=quinine tablets; PQ=primaquine; SP=sulfadoxine-pyrimethamine
